# Information and vaccine hesitancy: Evidence from the early stage of the vaccine roll-out in 28 European countries

**DOI:** 10.1371/journal.pone.0273555

**Published:** 2022-09-21

**Authors:** Francesca Agosti, Veronica Toffolutti, Nicolò Cavalli, Sanna Nivakoski, Massimiliano Mascherini, Arnstein Aassve

**Affiliations:** 1 “Carlo F. Dondena” Centre for Research on Social Dynamics and Public Policies, Bocconi University, Milano, Italy; 2 Wolfson Institute for Population Health – Centre for Evaluation and Methods, Queen Mary University of London, London, United Kingdom; 3 Department of Social and Political Science, Bocconi University, Milano, Italy; 4 Nuffield College, University of Oxford, Oxford, United Kingdom; 5 European Foundation for the Improvement of Living and Working Conditions, Dublin, Ireland; National Institute of Public Finance and Policy, INDIA

## Abstract

The success of mass vaccination programs against SARS-CoV-2 hinges on the public’s acceptance of the vaccines. During a vaccine roll-out, individuals have limited information about the potential side-effects and benefits. Given the public health concern of the COVID pandemic, providing appropriate information fast matters for the success of the campaign. In this paper, time-trends in vaccine hesitancy were examined using a sample of 35,390 respondents from the Eurofound’s Living, Working and COVID-19 (LWC) data collected between 12 February and 28 March 2021 across 28 European countries. The data cover the initial stage of the vaccine roll-out. We exploit the fact that during this period, news about rare cases of blood clots with low blood platelets were potentially linked to the Oxford/AstraZeneca vaccine (or Vaxzeveria). Multivariate regression models were used to analyze i) vaccine hesitancy trends, and whether any trend-change was associated with the link between the AstraZeneca vaccine ii) and blood clots (AstraZeneca controversy), and iii) the suspension among several European countries. Our estimates show that vaccine hesitancy increased over the early stage of the vaccine roll-out (0·002, 95% CI: [0·002 to 0·003]), a positive shift took place in the likelihood of hesitancy following the controversy (0·230, 95% CI: [0·157 to 0·302]), with the trend subsequently turning negative (-0·007, 95% CI: [-0·010 to -0·005]). Countries deciding to suspend the AstraZeneca vaccine experienced an increase in vaccine hesitancy after the suspensions (0·068, 95% CI: [0·04 to 0·095]). Trust in institutions is negatively associated with vaccine hesitancy. The results suggest that SARS-CoV-2 vaccine hesitancy increased steadily since the beginning of the vaccine roll-out and the AstraZeneca controversy and its suspension, made modest (though significant) contributions to increased hesitancy.

## Introduction

As of 3 November 2021, 220 countries and territories reported a total of 27,772,027 confirmed COVID-19 cases and a related death toll of 5,017,528 [1]. Europe has been hit hard by the pandemic with the UK, Russia, Germany, Romania in the top-10 globally [2] for both the number of confirmed cases and related deaths, and GDP loss exceeding 10% [3]. To curb the spread of the disease and to ease pressures on the already overwhelmed healthcare systems, most countries adopted restrictive non-pharmaceutical measures, such as school closures and lockdowns. However, the only long-term solution comes through the development of either safe and effective vaccine or an effective treatment for the disease itself. Currently, according to The *New York Times* Vaccine Tracker, there are 23 vaccines authorized or approved against the SARS-CoV-2 virus [4]. Four of them have been approved by the European Medicines Agency (EMA), namely BioNTech-Pfizer, Oxford/AstraZeneca (or Vaxzeveria), Moderna and Johnson&Johnson [5], with a coordinated vaccine roll-out beginning on 27 December 2020 [5].

Individuals’ willingness to get vaccinated plays a crucial role in achieving herd immunity [6]. With respect to COVID-19, vaccine uptake needs to be between approximately 67% and 80% to reduce spread of the disease [7, 8]. As the YouGov “COVID-19: willingness to be vaccinated” survey shows [9], vaccine hesitancy in European countries has decreased substantially, a fact reflected by current rates of actual vaccinations: as of 3 November 2021, 65·4% of the total European Union (EU) population are fully vaccinated. However, there are substantial differences among the EU countries: Portugal is the country with the highest uptake of full vaccination (81%), while in Bulgaria the share is as low as 21.9% [4]. To increase vaccination rates, many countries undertook strong measures. In July, the French prime minister, Emmanuel Macron, mandated proof of vaccination for entering shopping or leisure/culture centers, and for healthcare workers [10]. Since then, also other European countries, such as Austria, Belgium, Cyprus, Denmark, France, Germany, Greece, Hungary, Ireland, Italy, Luxembourg, the Netherlands, Portugal and Slovenia introduced some form of a COVID-19 pass [11]. Thus, despite great strides made, vaccine hesitancy remains an important issue.

Vaccine hesitancy is “a delay in acceptance or refusal of vaccination despite availability of vaccination services”, and is obviously driven by several factors, including doubts and worries about the safety of the vaccine [12]. The WHO EURO Vaccine Communications Working Group argues that vaccine hesitancy has three main determinants: complacency, convenience and confidence, the so-called “3 C Model” [12]. Complacency becomes an issue when the perceived risks related to the disease are low (and hence, vaccination is deemed not to be necessary). Convenience concerns physical availability, affordability, geographical accessibility and quality of the vaccination service. Confidence, meanwhile, is related to trust in: (i) the effectiveness and safety of vaccines; (ii) the system that delivers them (the healthcare system); and (iii) the reasons that push the government to start the vaccination campaign. Even before the coronavirus pandemic, WHO had already highlighted vaccine hesitancy as one of the ten leading threats to global health [13]. Therefore, understanding the causes of vaccine hesitancy is of paramount importance. Previous studies report that COVID-19 vaccine acceptancy increases with increasing age, education, and income level, and that vaccine acceptance is more likely among male individuals and among professionals, managers and teachers as compared to manual workers and farmers [14–22]. There are also substantial variations in vaccine acceptance across countries, including in settings with higher acceptance of other vaccinations [23–25]. COVID-19 related factors, such as perceived risk of COVID-19 infection, and having tested positive for COVID-19 in the past are associated with lower vaccine hesitancy [26, 27]. One of the strongest predictors of COVID-19 vaccination intentions is a person’s trust in the safety of the vaccine [28–31] and their concerns over adverse side effects of vaccination [30, 32]. The record time between the development and the roll-out of the vaccines against COVID-19 contributed to the concerns about vaccine safety [23, 33–38]. Additionally, the novelty of the mRNA vaccine might have sparked further suspicion and concern. It is also well established that there were substantial misinformation and conspiracy theories circulating in social media (often organized by anti-vaccination groups) [38–41] that could exacerbate hesitancy towards getting vaccinated [42]. A study conducted in eight European countries confirms these conjectures [33]: the major concerns about COVID-19 vaccines declared by respondents were the fear of side effects, the lack of evidence regarding the long-term effects of the vaccines, the speed with which the vaccines were developed and the distrust towards the government and pharmaceutical companies. Low levels of trust in the government and healthcare system was found to be a barrier to vaccine uptake also in other existing studies [43, 44] and distrust in science and government, as well as an unstable political situation, also play a major role in vaccine rejection [45, 46]. Similarly, vaccine acceptance is more likely among people who report a higher level of trust in information coming from government sources [25].

The present study focuses on the early phase of the vaccine roll-out, which includes the period when in many countries the AstraZeneca vaccine was pulled out of the vaccination program. This vaccine has been linked to very rare cases of blood clots with low blood platelets [47], but the small number of fatalities received considerable media attention. Over the second week of March 2021, several European countries paused, as a precautionary measure, inoculations using the AstraZeneca vaccine. As a result, on 11 March, the EMA opened formal investigations into 15 cases of deep-vein thrombosis (DVT) and 22 cases of pulmonary embolism–a blood clot that has entered the lungs–among those who had been vaccinated [48]. A specific batch of the AstraZeneca vaccine was consequently withdrawn. Suspensions began immediately following the EMA announcement. By 16 March 2021, 17 EU Member States, namely Austria, Bulgaria, Cyprus, Denmark, France, Germany, Ireland, Italy, Latvia, Lithuania, Luxembourg, the Netherlands, Portugal, Romania, Slovenia, Spain and Sweden had suspended the use of the vaccine (see S2 Appendix for the precise list of suspension dates) [49]. On 18 March, the EMA declared that the vaccine was safe to use and that its benefits outweighed the risks. Despite these assurances, trust in the safety of the AstraZeneca vaccine may have decreased as a result, with a potential knock-on effect on vaccine hesitancy more generally. Indeed, intensive media coverage of adverse events may have exacerbated concerns about the vaccine’s side effects [50]. As such the suspension of Vaxzeveria represent a natural experiment where we compare individuals living in countries where the vaccine was paused and hence did not have accesses to that said vaccine (the treated), and those living in the other countries which had continued access to the vaccine (controls). This can have important repercussions in terms of vaccine hesitancy. A YouGov poll carried out in February 2021 found that 43% of respondents in four European countries (Germany, France, Italy and Spain) believed that the AstraZeneca vaccine was unsafe. This share rose by 18 percentage points when the poll was carried out three weeks later, after the discovery of the first cases of blood clots. In Italy, the rise was of a magnitude of 27 percentage points, with 43% of Italians in the later survey believing the vaccine to be unsafe. Similar patterns were found in Spain and Germany [51, 52]. According to analysis of YouGov data from Britain, it is not clear if the blood clot issues surrounding the AstraZeneca vaccine had an effect on vaccine hesitancy [53], although concerns about the perceived safety increased immediately after controversy. A longitudinal study conducted in Denmark found that the reports of thromboembolic events in relation to the Oxford-AstraZeneca COVID-19 vaccine did not seem to affect vaccine willingness there [54]. Against this backdrop, this study investigates i) the overall impact of the AstraZeneca controversy on vaccine hesitancy in Europe in the period around the controversy and ii) the impact of AstraZeneca vaccine suspensions on vaccine hesitancy (at the country level). In the first part of the analysis, we use an OLS regression to examine the time trend of vaccine hesitancy during the first phase of the vaccine roll-out, which includes the period of the controversy (defined as the time period following 11 March 2021, when the first formal investigations were opened). In this part, data retrieved from the Google Trend platform are also analyzed. Secondly, we assess how a particular policy implementation, i.e. an individual country’s decision to suspend the AstraZeneca vaccine, affected vaccine hesitancy. To this end, a Difference-in-Differences (DiD) [55] model is used to compare vaccine hesitancy in suspending countries vis-à-vis those which continued the use of the AstraZeneca vaccine, controlling for time trends and observable individual-level characteristics.

## Materials and methods

The data for this study come from the third round of the LWC survey. It is a population-based online survey, the first round of which was initiated in April 2020. The data collection of the third round took place between 15 February and 30 March 2021, covering adults aged 18 and over living in Europe. The recruitment of the participants was carried out through social media (i.e. advertising through the Facebook platform), and had a certain element of snowball sampling since respondents could share the survey link to their facebook friends. The survey uses a structured questionnaire to investigate the impacts of the pandemic on living and working conditions. During the third round of the survey, respondents were also asked about their vaccination status and their likelihood to get vaccinated when given the opportunity. To the best of the authors’ knowledge, it is the only large-scale survey that provides EU-wide information about attitudes towards COVID-19 vaccination and coincides with the AstraZeneca vaccine controversy and suspensions. The majority of the survey questions were based on those used in the European Quality of Life Surveys (EQLS) [56] and the European Working Conditions Surveys (EWCS) [57].

The protocol for Eurofound’s Living, Working and COVID-19 survey was reviewed and approved both by Eurofound Management Advisory Committee and by Eurofound’s legal and data protection advisors. As a European agency, Eurofound is committed to ensuring that the research it conducts and coordinates complies with relevant regulatory and industry codes of practice, including data protection and other legal obligations in all European Member States. In line with these codes, standards and GDPR, specific attention was paid to: Consent for data collection, consent to send a customised report, consent to be re-contacted, opting out/data deletion, and secure storage of respondents’ data and pseudo-anonymization. Respondents were informed that their responses would be used solely for research purposes, and would be stored separately from personal information such as email address, which would be used to invite them to participate in the next round of the survey if consent was given. They were informed that their participation, together with their individual responses to the questions, would be kept strictly confidential implying anonymity of all respondents in the research results (containing statistical information only). Survey participants received a description of the study and could decline to participate or withdraw at any time, and were provided with the option “I don’t want to respond” for all questions. The authors did not obtain any personal information about the participants. Participants’ responses were treated confidentially, and anonymous responses were utilized for the analyses presented herein. Please note that we have also uploaded a separate document (S1 File) which is the informed consent form presented to the survey participants.

The vaccine hesitancy was measured by the question “How likely or unlikely is it that you will take the COVID-19 vaccine when it becomes available to you?”. Only non-vaccinated individuals were administrated the above question. Answers were recorded on a 5-point scale, ranging from “very likely” to “very unlikely”. We define vaccine hesitancy using a binary indicator variable: an individual is defined as hesitant if they answer “rather unlikely” or “very unlikely”, and non-hesitant otherwise.

Respondents’ trust in various institutions was recorded with the question “Please tell me how much you personally trust each of the following institutions”, with questions for categories including: “the healthcare system”, “your country’s government”, “the European Union” and “pharmaceutical firms”. The answers were recorded on a 10-point scale, with a range from 1 (“do not trust at all”) to 10 (“trust completely”). S4 Appendix reports summary statistics for the main dependent and independent variables that will be used in the analysis.

**Fig 1:**
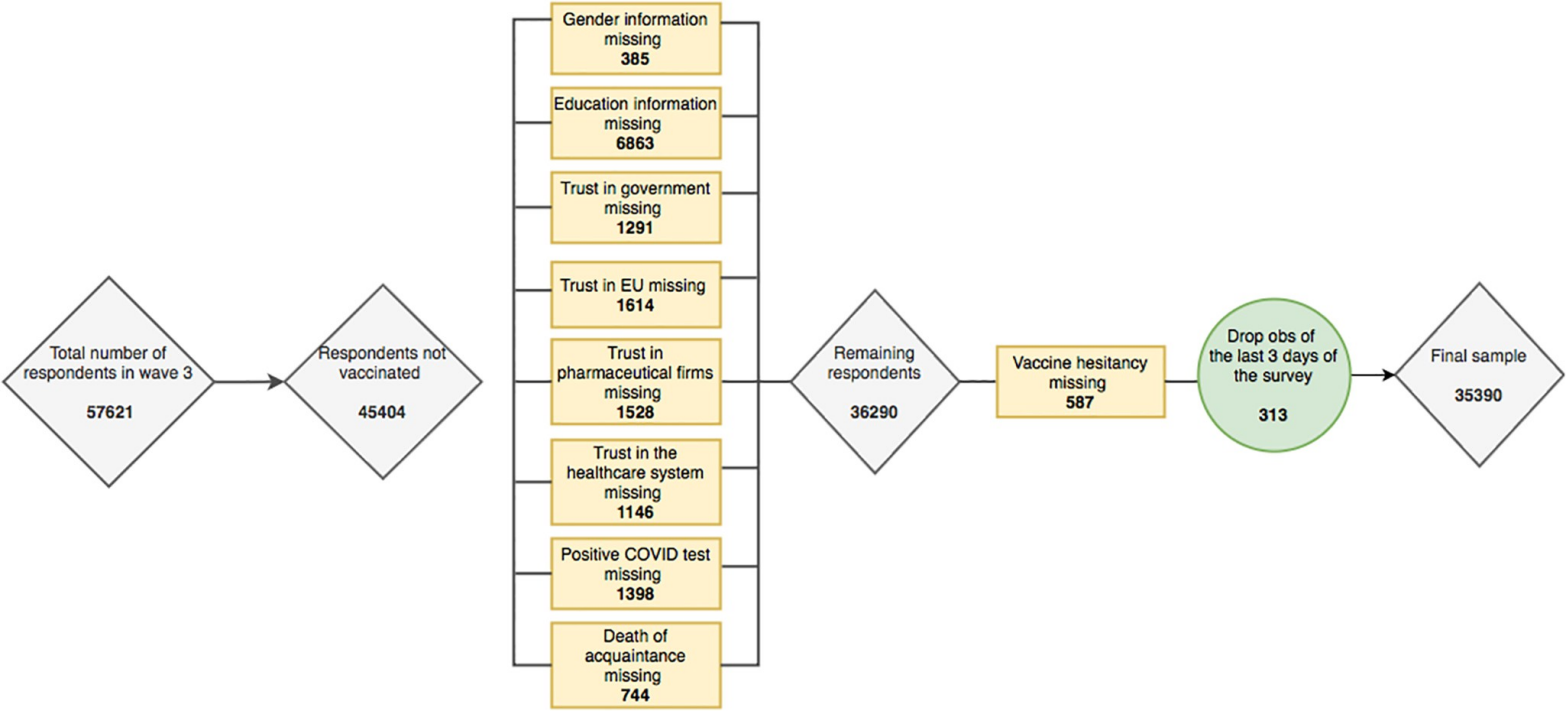
Sample selection. Notes: The initial sample of 57,621 respondents is obtained by eliminating those individuals who declare themselves to be older than 100 or who have missing age information.

Fig 1 presents the sample selection process. 57,621 Europeans were interviewed in the third LWC round. Individuals who reported to have received at least one dose of a COVID-19 vaccine (7·87% of the respondents of the third round, see S3 Appendix) and those for whom this information was absent (13·33%) were excluded, as they were not asked the vaccine hesitancy question. A further 9,114 individuals were excluded as they reported missing values in key variables. 587 respondents did not answer the vaccine hesitancy question and were thus also excluded from the analysis. Lastly, we excluded the last three days of the survey (29–31 March), corresponding to 313 observations, as only a few interviews, with a large percentage of outliers in their observable characteristics, were completed on those days. Our final sample consists of 35,390 individuals from 28 countries: the 27 EU Member States and the U.K.

### Time trend in vaccine hesitancy

**Fig 2:**
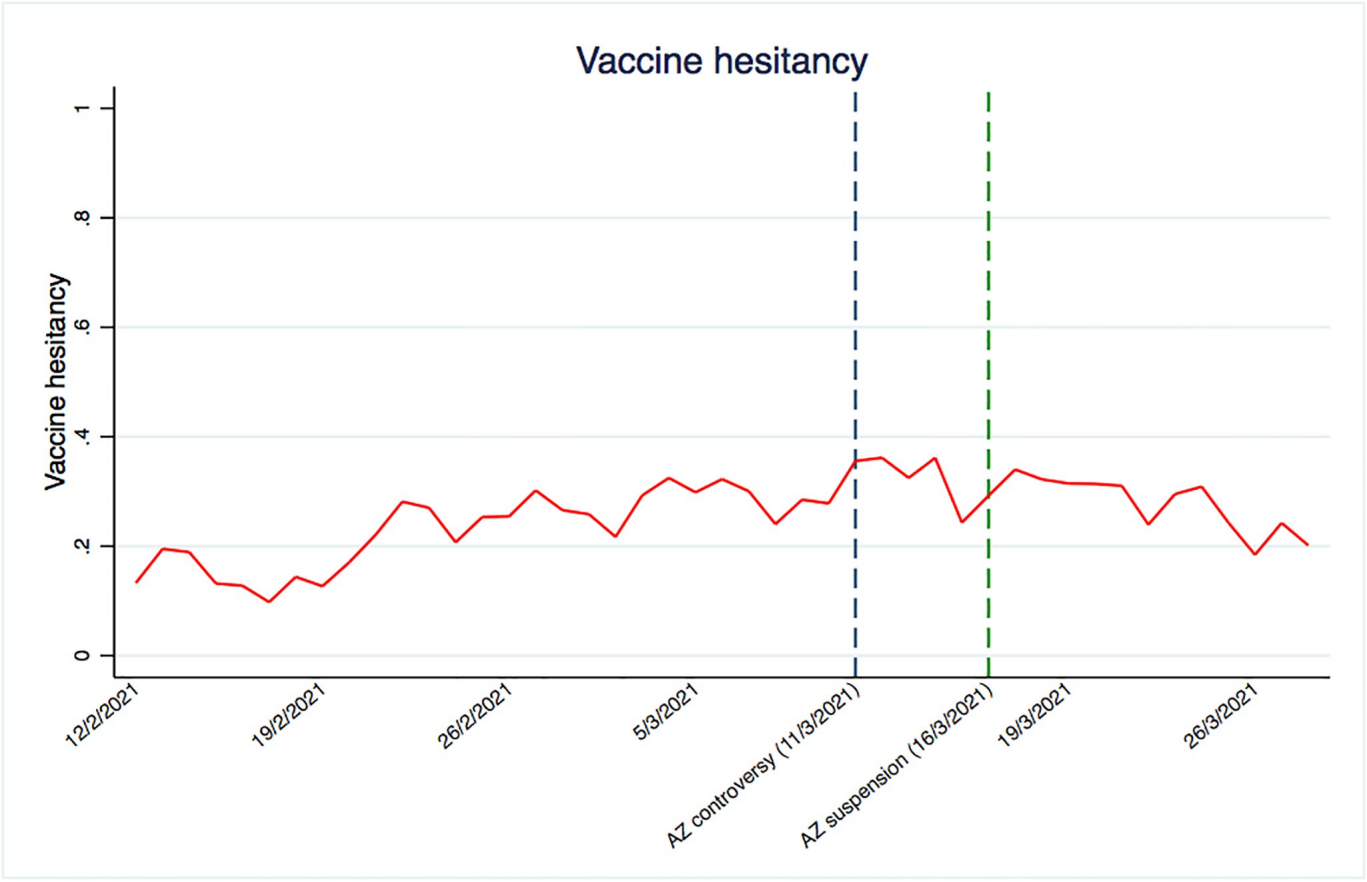
Vaccine hesitancy time-trend. Notes: the graph shows the time-trend of the vaccine hesitancy during the period from 12 February 2021 to 28 Marrh 2021 The blue dashed line represents the moment of the AstraZeneca controversy, coinciding with 11 March 2021, while the green dashed line represents the date when the AstraZeneca vaccine was suspended across a number of countries (16 March 2021).

Fig 2 presents the time-trend of the vaccine hesitancy during the survey period. Intuitively, one would expect vaccine hesitancy to decline as people observe the roll-out of the vaccine, and as more information becomes available to them, such as the curb on the number of cases of infection. In Fig 2, the blue dashed line indicates the start of AstraZeneca controversy, while the green dashed line represents the date when the AstraZeneca vaccine was suspended across a number of countries.

**Fig 3:**
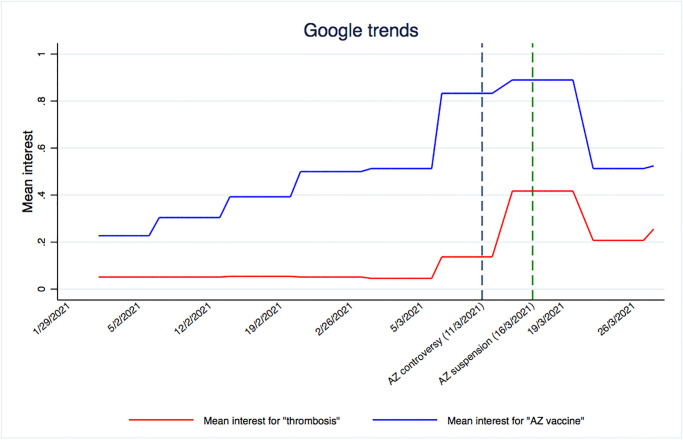
Google trends in the search of “AstraZeneca vaccine” and “thrombosis”. Notes: the graph presents the mean interest for two keywords, "thrombosis" and "AstraZeneca vaccine", during the period from 1 February 2021 to 21 March 2021. Search volumes for these keywords have been retrieved from Google Trends in seven languages: English, French, German, Italian, Spanish, Polisch and Danish.

We see a rise in vaccine hesitancy up to the suspension date and then a decline. One possible driver behind this upwards trend is that the public is learning about the efficacy of the vaccine, but also about its potential side effects. We compare the vaccine hesitancy trend with trends from the Google search engine, retrieved from Google Trends to provide qualitative evidence that interest towards both AstraZeneca and thrombosis rose together at the same time as the controversy broke. Whereas we do not claim that this trend in search volumes is reflective of lower trust in vaccine, Google Trends provides a grasp of the responsiveness of populations to the ’controversy’. Fig 3 shows the trends over time for two sets of keywords: “AstraZeneca vaccine”, and the most known side effect, “thrombosis”. Search volumes for these keywords have been retrieved in seven languages: English, French, German, Italian, Spanish, Polish and Danish, which accounts for 80% of the European population. The pattern in Fig 3 suggests that searches for the vaccine increased over time in a similar pattern as vaccine hesitancy. In parallel, there is also a sharp increase in searches for the keyword “thrombosis”. Although this happens with some delay compared to the trends associated with the AstraZeneca vaccine, the trend more than doubles in the lead-up to the AstraZeneca suspension. Although here we present an average across countries, all countries individually show very similar trends.

These qualitative findings are consistent with another study from Italy, showing that search trends for both “AstraZeneca vaccine” and “thrombosis” increased from 11 February to 1 April [58]. Interestingly, the spike in the Google search volumes appears around the suspension of the AstraZeneca vaccine, and then subsides. This is consistent with Fig 2, where we have shown that the trend in vaccine hesitancy flattens out after the AstraZeneca suspension.

## Results

### Effect of the AstraZeneca controversy on vaccine hesitancy

**Fig 4:**
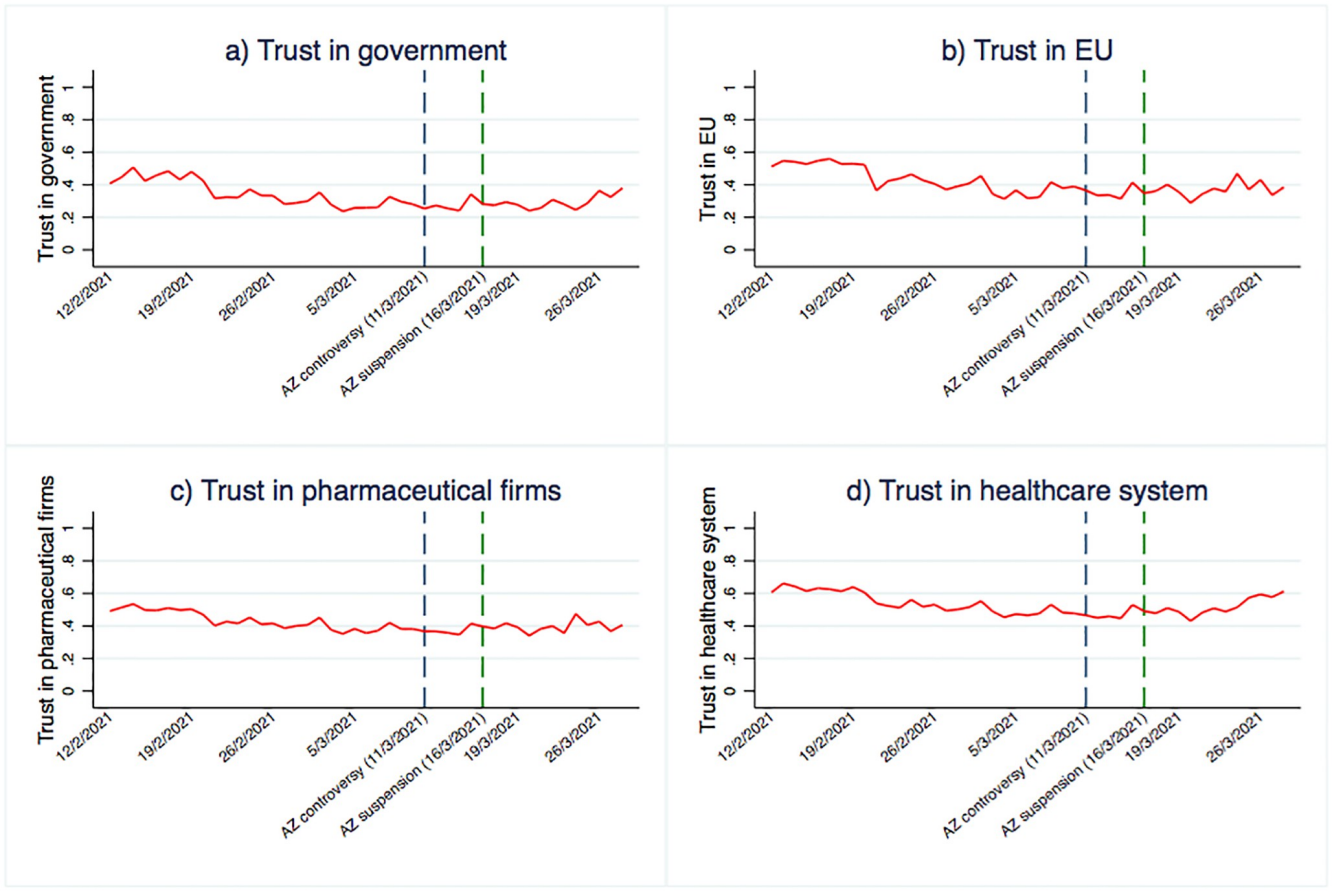
Time-trend in Trust in a) Government b) The EU c) Pharmaceutical Companies and d) Healthcare Systems. Notes: the graphs show the time trend of four trust measures (trust in government (a), trust in EU (b), trust in pharmaceutical firms (c), trust in the healthcare system (d)) during the period from 12 February 2021 to 28 March 2021. The blue dashed lines represent the moment of the AstraZeneca controversy, coinciding with 11 March 2021, while the green dashed lines represent the date when the AstraZeneca vaccine was suspended across a number of countries (16 March 2021).

Confidence is a central tenet of the “3C model” of vaccine hesitancy. The LWC survey includes questions regarding trust in the systems that are responsible for the development and delivery of the vaccines. Fig 4 presents trends of trust in a) the national government (which coordinated and promoted the vaccine); b) the EU (which decided which vaccines to authorise and how to distribute them); c) pharmaceutical firms (that produced the vaccines); and d) the healthcare system (responsible for the distribution of vaccines). As we can see, the four trust measures follow a similar pattern over time. Trust decreases and reaches its lowest levels a few days before the suspension of the AstraZeneca vaccine. Thereafter, trust in these four entities either flattens out or slightly increases. This is again consistent with the trend in vaccine hesitancy and the Google search keywords discussed above (Fig 3).

To study if the AstraZeneca controversy is associated with changes in vaccine hesitancy, we use a simple OLS regression model described below in Eq 1,

$$VH_{itc}=\alpha +\beta_{1}Trend_{it}+\beta_{2}AZControversy_{t}+\beta_{3}AZControversy_{t}*Trend_{it}+\gamma^{\prime }X_{ic}+\delta^{\prime }Trust_{ic}+\epsilon_{ict}$$ (1)

where *vaccine hesitancy* (VH) represents a dummy variable equal to 1 if respondent *i* living in country *c* at time *t* is vaccine hesitant. Overall, in our sample, 22·8% of respondents declare to be vaccine hesitant. *Trend* is a continuous variable equal to the day of interview. *AZControversy* is a dummy variable that takes value 1 since 11 March 2021, and 0 before. This date marks the beginning of the EMA formal investigation on the possible sides effect of the Vaxzevria [59]. *X* is a vector representing a set of individual characteristics, namely: age in groups (18–24, 25–34, 35–44, 45–54, 55+); gender; if the respondent holds a tertiary education degree; tested positive for COVID-19 since the outbreak of the pandemic in early 2020; and had at least one acquaintance who died of COVID-19. In Eq (1), trust represents the self-reported levels of trust in the national government, the EU, the healthcare system, and pharmaceutical firms. *ϵ* represents a random error term. *β*_1_ represents the coefficient capturing the time trend in vaccine hesitancy, whereas *β*_2_ captures the change in levels of vaccine hesitancy after the AstraZeneca controversy. It consequently estimates discontinuities in vaccine hesitancy associated with the controversy. Finally, *β*_3_ measures any change in the vaccine hesitancy trend after the AstraZeneca controversy. STATA 16 was used in the estimation. We employed robust standard errors with the command *robust*.

Table 1 presents the results. In Column 1, besides controlling for the covariates (*X*), the main explanatory variable is the time-trend. Column 2 includes a dummy variable indicating whether the individual was interviewed after (or before) the AstraZeneca controversy. Column 3 includes the interaction between the time-trend variable and the AstraZeneca controversy. Finally, in column 4, we also include the level of trust across four main institutions: the national government, the EU, the healthcare system, and pharmaceutical companies.

**Table 1:** Vaccine hesitancy trend results.

	(1)	(2)	(3)	(4)
VARIABLES	vaccine hesitancy	vaccine hesitancy	vaccine hesitancy	vaccine hesitancy
**Trend**	0.004***	0.007***	0.008***	0.002***
	(0.004–0.005)	(0.006–0.007)	(0.007–0.008)	(0.002–0.003)
**AstraZeneca controversy**		-0.075***	0.413***	0.230***
		(-0.094–-0.055)	(0.330–0.496)	(0.157–0.302)
**Trend** * **AstraZeneca controversy**			-0.015***	-0.007***
			(-0.017–-0.012)	(-0.010–-0.005)
**Age group (Ref:25–34)**				
18–24	-0.064***	-0.061***	-0.052***	0.004
	(-0.090–-0.038)	(-0.087–-0.036)	(-0.077–-0.026)	(-0.019–0.027)
35–44	0.004	0.002	-0.000	-0.029***
	(-0.013–0.021)	(-0.014–0.019)	(-0.017–0.017)	(-0.044–-0.014)
45–54	0.011	0.009	0.005	-0.040***
	(-0.005–0.028)	(-0.008–0.025)	(-0.011–0.022)	(-0.054–-0.026)
> = 55	-0.035***	-0.039***	-0.043***	-0.077***
	(-0.049–-0.020)	(-0.053–-0.024)	(-0.058–-0.028)	(-0.090–-0.064)
**Female**	-0.022***	-0.022***	-0.019***	-0.010**
	(-0.031–-0.013)	(-0.031–-0.013)	(-0.028–-0.010)	(-0.018–-0.002)
**Tertiary education**	-0.104***	-0.103***	-0.102***	-0.055***
	(-0.114–-0.094)	(-0.113–-0.093)	(-0.112–-0.092)	(-0.063–-0.046)
**Tested positive to COVID-19**	0.033***	0.034***	0.036***	0.027***
	(0.016–0.051)	(0.017–0.051)	(0.019–0.053)	(0.012–0.043)
**Death of acquaintance**	-0.067***	-0.067***	-0.066***	-0.057***
	(-0.080–-0.053)	(-0.080–-0.053)	(-0.079–-0.053)	(-0.070–-0.045)
**Trust in the government**				-0.010***
				(-0.012–-0.008)
**Trust in the EU**				-0.020***
				(-0.022–-0.018)
**Trust in the healthcare system**				-0.017***
				(-0.019–-0.015)
**Trust in pharmaceutical firms**				-0.044***
				(-0.046–-0.042)
Constant	0.255***	0.236***	0.219***	0.742***
	(0.237–0.273)	(0.218–0.254)	(0.201–0.237)	(0.721–0.762)
Observations	35,390	35,390	35,390	35,390
R-squared	0.036	0.038	0.042	0.253

Notes: Estimation results from the Eq 1. Data come from the third wave of the Eurofound “Living, Working and COVID-19”. The outcome variable represents a dummy variable equal to 1 if the individual is (rather) unlikely to get vaccinated if he or she was offered the vaccine against COVID-19 and 0 otherwise. Trend is a continuous variable equal to the day of interview. AZControversy is a dummy variable that takes value 1 since 11 March 2021 (date of the controversy), and 0 before. Robust standard errors are employed. 95% confidence intervals are presented in parentheses.

*** p<0.01,

** p<0.05,

* p<0.1.

**Fig 5:**
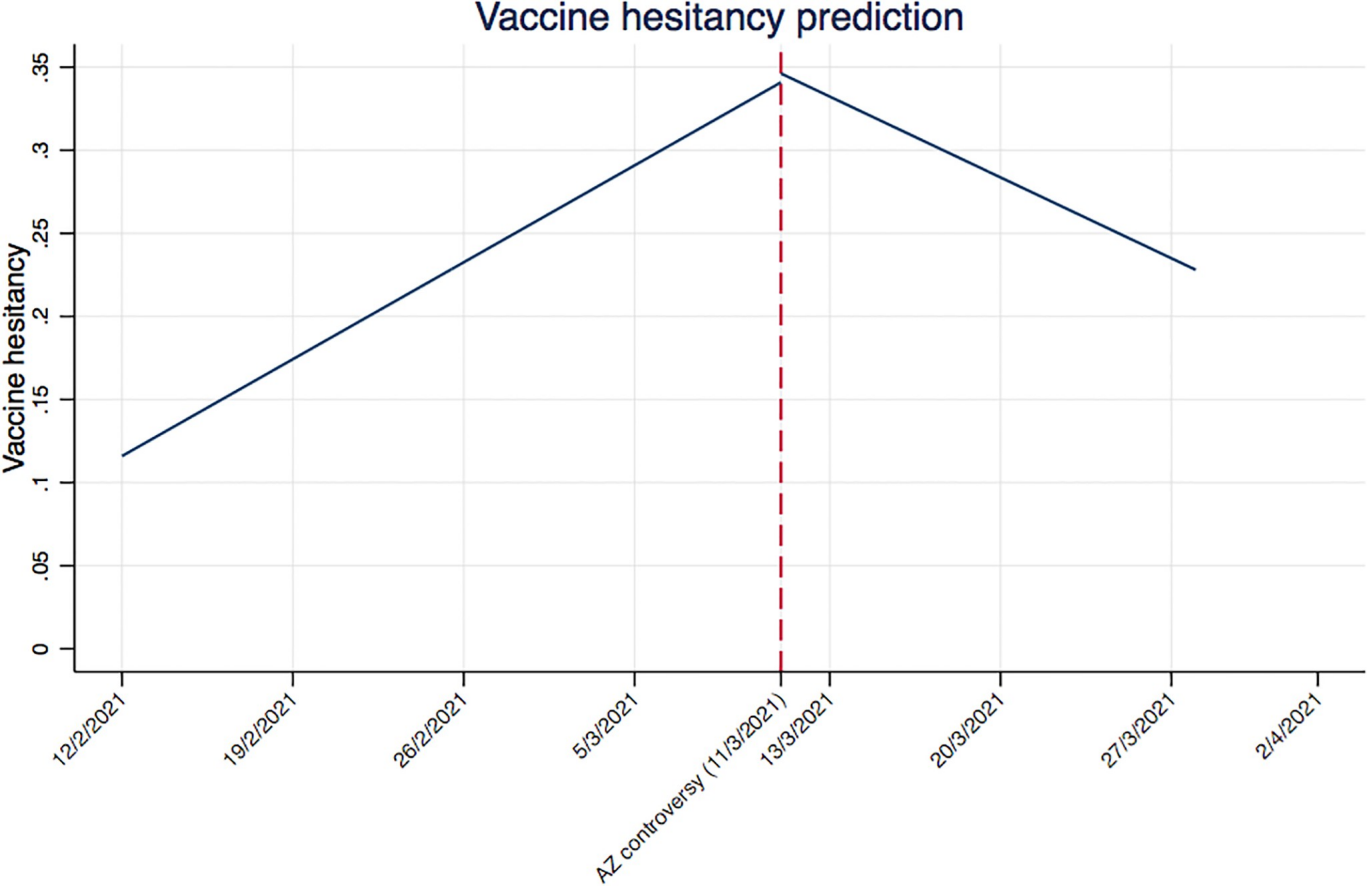
Linear prediction vaccine hesitancy. Notes: the graph presents the linear prediction of vaccine hesitancy. The red dashed line coincides with the date of the AstraZeneca controversy (11 March 2021).

Consistent with our descriptive analysis, estimates show that vaccine hesitancy increase over time; more specifically, the coefficient *β*_1_, equal to 0·004 (95% CI: [0·004 to 0·005]) represents the rise in vaccine hesitancy over time (column 1). In column 2, this effect increases to 0·007 (95% CI: [0·006 to 0·007]) when we include the AstraZeneca controversy indicator, which appears to significantly decrease vaccine hesitancy, by about 0·075 [95% CI: (-0·094 to -0·055)]. When we add the interaction between the trend and the controversy dummy (column 3), the vaccine hesitancy trend remains positive and slightly increases (0·008, 95% CI: [0·007 to 0·008]). The interaction itself is negative (-0·015 [95% CI: -0·017 to -0·012]) suggesting a change in slope (from positive to negative) of the trend in vaccine hesitancy after the AstraZeneca controversy. When this change in trend is considered, the coefficient capturing the effect of the AstraZeneca controversy becomes positive, suggesting a higher probability of observing vaccine hesitant respondents after the event. In the fourth model (column 4) we add the four trust variables. They all have negative and statistically significant coefficients, suggesting that trust is negatively associated with vaccine hesitancy. Out of the four measures, trust in pharmaceutical firms has the largest negative coefficient (-0·044, 95% CI: [-0·046 to -0·042]), whereas trust in national government is the smallest (-0·010, 95% CI: [-0·012 to -0·008]). However, the overall pattern of an increasing trend prior to the AstraZeneca controversy (0·002, 95% CI: [0·002 to 0·003]), a shift in the level (0·230, 95% CI: [0·157 to 0·302]), with the trend subsequently turning negative (-0·007, 95% CI: [-0·010 to -0·005]) remains after the trust variables are included. It is important to note that drawing a direct comparison between the size of *β*1, *β*2 and *β*3 coefficients is difficult because they tend to move at different speed and also because the first is a continuous variable while the latter are binary variables. To better illustrate the effect of the controversy, we use the estimated model to predict vaccine hesitance over the period, including the 11^th^ March and the period after. Fig 5 plot the estimated pattern from this model, which shows a steady increase in vaccine hesitancy up to the AstraZeneca controversy and then a downward trend.

Both *β*1 and *β*2 point towards a temporal increase in vaccine hesitancy, whereas the negative sign of *β*3 gives the negative trend following the date of the controversy.

To test the association between controversy, trust and vaccine hesitancy we also carry out a mediation analysis which confirms the role of the trust as mediator. The results are reported in the robustness check section in S5 Appendix.

### Did the AstraZeneca suspensions affect vaccine hesitancy?

It is plausible that the AstraZeneca controversy played a role in the evolution of vaccine hesitancy in Europe in the period considered. Blood clots or fatality cases stemming from the AstraZeneca vaccine, would presumably increase vaccine hesitancy and by extension, decrease trust in pharmaceutical companies. But as we have seen, the time-trend suggests otherwise. Vaccine hesitancy was steadily rising well before 16 March (the suspension date), and, as Fig 5 shows, the discontinuity around the date of the AstraZeneca controversy is modest (although statistically significant). Moreover, vaccine hesitancy appears to decline as the controversy evolves. Trust-trends present similar patterns. On the one hand, this begs the question whether the AstraZeneca suspension actually lowered vaccine hesitancy. This is the case if individuals reacted positively to the fact that institutions acted responsibly by suspending the vaccine. On the other hand, one could also argue that any suspension would confirm individuals’ suspicions, thereby driving up vaccine hesitancy among the public.

To investigate the mechanism, we use a Difference-in-Differences (DID) approach [55, 60], indicated in Eq 2 below. The idea behind its estimation is to compare the difference in the outcome before and after the exogenous event for both the treated and the controls and take its net variation. As both groups are subject to the same macro-economic trends the net effect should provide an unbiased estimation of the impact of the event–in this case the impact of the controversy. In our case, we split the countries into two groups: the treated group (Treated) composed of respondents from the 17 countries which governments decided to suspend the AstraZeneca vaccine, while the control group is made up of individuals from the remaining 11 countries (see appendix for more details). To identify the suspension we use a dummy variable (Suspension) which takes value 1 after March 16^th^ (the date when the bulk of countries suspended the AstraZeneca vaccine, and the peak in the coverage of the controversy in news media) and 0 otherwise. We are interested in the coefficient corresponding to the interaction between these two variables (*DD*_3_), which indicates the change in vaccine hesitancy after the suspension for countries that suspended the AstraZeneca vaccine, compared to the group of countries that did not. To control for macro-economic trends such as seasonality we adjust for months fixed-effects. However, this kind of model assumes that the only difference between the countries that withdrew the vaccines and those which carried on inoculations is due to the suspension itself. In other words, the model assumes that, prior to the suspension, the two sets of countries were exposed to similar trends in vaccine hesitancy. The parallel trend assumption is formally tested in the S1 Appendix [Graph A.1 in S1 Appendix], showing that we find no systematic difference in vaccine hesitancy trends prior to the suspension date of 16 March. The attentive reader will spot the similarities with Eq (1) above. However, Eq 1 cannot be defined as DID approach as there is no clear treatment group.


$$VH_{itc}=\alpha +DD_{1}Treated_{it}+DD_{2}Suspension_{t}+DD_{3}Suspension_{t}*Treated_{it}+\eta^{\prime }X_{ic}+\theta^{\prime }Trust_{ic}+\mu_{ict}$$ (2)

Table 2 compares the two sets of countries across the covariates used in the previous specification, with evidence of significant differences across all dimensions. Including these covariates in the DID model allows for the differences between the two groups to be controlled for.

**Table 2:** Differences between treated and control group.

VARIABLES	Control	Treated	Difference
**Age**	51.70	50.81	0.891***
			(0.152)
**Female**	0.619	0.637	-0.018***
			(0.005)
**Tertiary education**	0.623	0.694	-0.071***
			(0.005)
**Trust in government**	3.484	4.538	-1.054***
			(0.031)
**Trust in EU**	4.574	5.155	-0.581***
			(0.030)
**Trust in pharmaceutical firms**	4.739	5.002	-0.263***
			(0.027)
**Trust in healthcare system**	5.573	6.107	-0.533***
			(0.028)
**Tested positive to COVID-19**	0.067	0.079	-0.012***
			(0.003)
**Death of acquaintance**	0.084	0.104	-0.020***
			(0.003)
**Vaccine hesitancy**	0.235	0.224	0.010*
			(0.005)
**N**	14,463	20,927	35,390

Notes: Data come from the third wave of the Eurofound “Living, Working and COVID-19”. The table presents the differences between treated and control groups for 9 variables used as controls and for the outcome variable, vaccine hesitancy. Standard errors are presented in parentheses.

*** p<0.01,

** p<0.05,

* p<0.1.

The results of the DID analysis are reported in Table 3. Column 1 presents the results of the basic model that only includes the treatment indicator, the time variables and their interaction. In column 2, we also include socio-demographic variables: age group, gender and education. In the model in column 3, trust variables are added, while the last column presents the results of the most complete model, also including the two COVID-19-related variables (having been tested positive for COVID-19 and having experienced a COVID-19 related death of an acquaintance). In the model presented in column 2, the DID coefficient, our coefficient of interest, is 0·068 (95% CI: [0·04 to 0·095]). This estimate suggests that countries deciding to suspend the AstraZeneca vaccine, experienced an increase in vaccine hesitancy after the suspensions. In other words, it appears that suspending the AstraZeneca vaccine gave an impetus to vaccine hesitancy. In the next model (column 3), we also include variables capturing respondents’ reported trust levels. In this model, the DID coefficient remains positive and statistically significant (0·055, 95% CI: [0·030 to 0·079), suggesting that our result is robust even when controlling for the level of trust. In the last model, column 4, we add two additional control variables (related to personal experience with COVID-19), with estimates unchanged. In this model, countries that retracted the vaccine had a 5%-points higher level of vaccine hesitancy after the suspension, compared to countries that did not.

**Table 3:** DID results.

	(1)	(2)	(3)	(4)
VARIABLES	vaccine hesitancy	vaccine hesitancy	vaccine hesitancy	vaccine hesitancy
**Suspension moment**	0.024**	0.021**	-0.016*	-0.015*
	(0.004–0.044)	(0.002–0.041)	(-0.034–0.002)	(-0.033–0.003)
**Treated**	-0.018***	-0.009*	0.031***	0.032***
	(-0.027–-0.008)	(-0.018–0.001)	(0.022–0.039)	(0.023–0.040)
**Treated x suspension moment**	0.075***	0.068***	0.055***	0.053***
	(0.047–0.103)	(0.040–0.095)	(0.030–0.079)	(0.029–0.077)
**Age group (Ref: 25–34)**				
18–24		-0.050***	0.005	0.004
		(-0.076–-0.025)	(-0.018–0.027)	(-0.019–0.026)
35–44		0.005	-0.026***	-0.026***
		(-0.012–0.022)	(-0.041–-0.011)	(-0.040–-0.011)
45–54		0.015*	-0.035***	-0.034***
		(-0.002–0.031)	(-0.049–-0.021)	(-0.048–-0.019)
> = 55		-0.032***	-0.072***	-0.070***
		(-0.047–-0.017)	(-0.085–-0.059)	(-0.083–-0.057)
**Female**		-0.031***	-0.015***	-0.014***
		(-0.040–-0.022)	(-0.023–-0.007)	(-0.022–-0.006)
**Tertiary education**		-0.111***	-0.059***	-0.058***
		(-0.121–-0.101)	(-0.068–-0.050)	(-0.067–-0.049)
**Trust in the government**			-0.011***	-0.011***
			(-0.013–-0.010)	(-0.013–-0.010)
**Trust in the EU**			-0.021***	-0.021***
			(-0.023–-0.019)	(-0.023–-0.019)
**Trust in the healthcare system**			-0.018***	-0.018***
			(-0.020–-0.015)	(-0.020–-0.015)
**Trust in pharmaceutical firms**			-0.043***	-0.043***
			(-0.045–-0.041)	(-0.045–-0.041)
**Tested positive to COVID-19**				0.024***
				(0.009–0.040)
**Death of acquaintance**				-0.060***
				(-0.072–-0.047)
Constant	0.231***	0.332***	0.769***	0.769***
	(0.224–0.238)	(0.314–0.350)	(0.750–0.788)	(0.750–0.788)
Observations	35,390	35,390	35,390	35,390
R-squared	0.004	0.022	0.252	0.253

Notes: Estimation results from a DID model presented in Eq 2, where the treated (controls) represents individuals living in a country where the AstraZeneca vaccine was (NOT) suspended by 16 March. Suspension moment is a dummy variable that takes value 1 since 16 March 2021 (date of the first suspensions), and 0 before. 95% confidence intervals are presented in parentheses.

*** p<0.01,

** p<0.05,

* p<0.1.

## Discussion

This study investigates vaccine hesitancy during the early phase of the SARS-CoV-2 vaccine roll-out in Europe, which includes the AstraZeneca controversy, and its suspension in several countries. On the one hand, we assess whether the controversy itself affected vaccine hesitancy. On the other hand, we examine whether the *retraction* of the AstraZeneca vaccine affected vaccine hesitancy. To this end, we use individual level data for over 35,000 people from 28 European countries from the third wave of Eurofound’s LWC survey, which sampled individuals between 12 February and 28 March 2021. The period of this survey usefully overlapped with the suspension, across 17 European countries, of the AstraZeneca vaccine.

The results suggest that vaccine hesitancy grew steadily from the very beginning of the vaccine roll-out. Whereas the AstraZeneca controversy had an effect, it was a relatively small contribution to overall hesitancy. Instead, the data points to increased awareness of the AstraZeneca vaccine. As citizens start to realize that they may soon have the opportunity to get the vaccine, they also sought out information on its potential side effects and its safety. Here it is possible that individuals were more strongly affected by negative narratives, possibly communicated through the media, where suspected cases of severe side effects, even fatalities, were reported: with it, we also see a gradual decline in trust in the relevant institutions. In the case of pharmaceutical companies and government, the decline in trust was stronger after the suspension of the vaccine. Decline in trust and increased vaccine hesitancy appear to go hand in hand, as the mediation analysis demonstrated (see S5 Appendix). This is in stark contrast to an objective assessment of the risk. Given the sheer number of vaccinated individuals, the risk of life-threatening thrombosis is, based on reported cases, extremely small. Again, negative narratives of a small number of cases appear to trump any objective judgements of the undeniable positive public health effects from ending the pandemic.

Before proceeding with the policy implications, we should notice that this work has limitations. First, we study the willingness to be vaccinated rather than actually being (or not being) vaccinated. Second, our sample has been recruited online and trough a certain element of snowball sampling and is thus not fully representative of the countries included in the study. However, this kind of sampling strategy has been extensively used to assess COVID-19 vaccine hesitancy [61, 62]. Here, in particular, the sample contains a higher proportion of highly educated respondents, who tend to be more hesitant. Our results may consequently be on the conservative side. Third, there is some small variation in the suspension date across countries; therefore, we believe that our results are conservative as they represent an average across countries and do not capture the immediate reaction due to the suspension. Fourth, we look specifically at the AstraZeneca suspension, but we cannot distinguish to what extent there is hesitancy towards other manufacturers’ vaccines.

Our findings have nevertheless important implications. They show that the AstraZeneca controversy did not increase vaccine hesitancy among Europeans, suggesting that negative news coverage of the fatalities associated with the AstraZeneca vaccine did not affect vaccine hesitancy as much as one may have thought. Our results show instead that during the early phase of the roll-out of the vaccination program, there was a general rise in vaccine hesitancy. This result is coupled with a negative correlation between vaccine hesitancy and trust measures. With the assumption that the level of trust in the institutions that develop, distribute and deliver vaccines is reflected in the survey trust measures, these two results suggest that to decrease vaccine hesitancy in the early stage of the vaccine rollout, policy makers must engage in a clear, transparent and continuous communication about the vaccines and their potential side effects [56]. After all, it has been demonstrated that transparency sustains trust in health authorities and hinders the spread of conspiracy beliefs, without necessarily reducing intentions to be vaccinated [63, 64]. This is in line with recent findings for the UK, where vaccine hesitancy was higher–almost twice higher–for those who felt they have no say in government or who do not trust public officials [65]. How to raise trust? Recent evidence from the OECD suggest that engaging with citizens in consultations and focus groups concerning–in this case–vaccination might lead to a higher uptake. In parallel, speculations suggest that high ethical standards for public figures ads public institutions during emergency is vital to increase citizens’ trust [66].

As we have demonstrated, the public makes considerable efforts on their own to investigate possible side effects once they realize that they are about to receive the vaccine. Finally, the fact that the retraction of the AstraZeneca vaccine increased hesitancy introduces a dilemma for policy makers. Suspending the vaccine was in this case argued for on the grounds of public health concerns. Yet, at the same time, the very same decision increased hesitancy, which equally poses a threat to public health. With the data used in this analysis, we cannot proceed with a full cost-benefit analysis to investigate on the optimal policy choice. Still, our analysis does demonstrate that suspending a vaccine in the manner of the AstraZeneca case, imposes an important externality through increased vaccine hesitancy. This is clearly an area for future research. Notwithstanding these limitations, the fact that recent speculation suggests that ‘the harm to AstraZeneca jab’s reputation probably killed thousands’ should be considered a reason for considerable concern [67].

## Supporting information

S1 Appendix Parallel trend assumption. (PDF)

S2 Appendix List of suspension dates in the 17 countries. (PDF)

S3 Appendix Percentage of respondents who received at least one dose of a vaccine, by country. (PDF)

S4 Appendix Summary statistics of key variables. (PDF)

S5 Appendix Mediation analysis. (PDF)

S1 File Data protection notice on the COVID-19 survey. (PDF)
