# Supplementary material for: Information and vaccine hesitancy: Evidence from the early stage of the vaccine roll-out in 28 European countries
Source: PLoS One. 2022 Sep 21;17(9):e0273555. doi: 10.1371/journal.pone.0273555 (PMC9491558; doi:10.1371/journal.pone.0273555)
Supplement: S1 Appendix — (PDF) [file pone.0273555.s001.pdf]

S1 Appendix – Parallel trend assumption

The difference-in-difference (DID) technique is typically used to estimate the effect of a specific event, by comparing the changes in outcomes over time between the treatment and the control group. The DID design uses data from both groups to obtain an appropriate counterfactual with the goal of making causal interpretation. However, a critical requirement is that prior to the event, treatment and control group should have parallel trends in terms of the outcome. Violation of this assumption leads to bias and compromises any causal interpretation. The parallel trend assumption can be checked through visual inspection. Figure A.1 depicts the two trends for the treated and the control group before the suspension of the AstraZeneca vaccine (our treatment).

Figure A.1: Parallel trend assumption

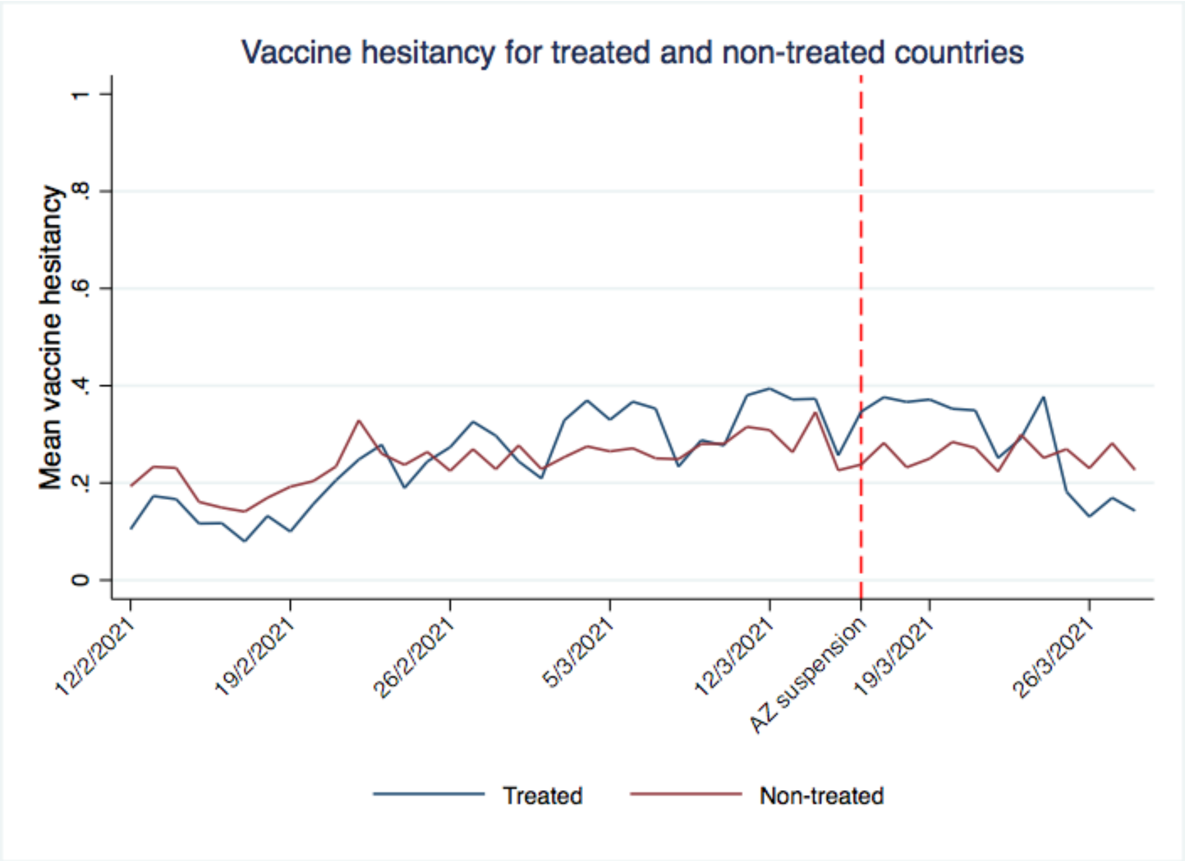

Whereas the trends are similar overall, we take note that the trend for those who have been treated fluctuates more. Because of this, we further test the assumption with the help of regressions, but restricted to set time intervals. The model we use is described by equation (A1):

$$VH_{itc} = \alpha + \beta_1 Trend_{it} + \beta_2 Treated_t + \beta_3 Treated_t * Trend_{it} + \gamma' X_{ic} + \epsilon_{ict} \quad \text{(A1)}$$

where *Trend* is a continuous variable equal to the day when the individuals were interviewed, *Treated* is a dummy equal to 1 if the individual lives in a country which suspended the AstraZeneca vaccine and 0 otherwise. *X* represents a set of individual characteristics such as gender, age, education, having tested positive to COVID-19 and know someone who died because of coronavirus.

We are interested on the coefficient of the interaction between *Treated* and *Trend* since this would indicate a deviation from the parallel trend assumption. In Table A.1, we present the estimates obtained through equation (A1) for 5 segments of the trend. We can see that the coefficient corresponding to the interaction is significant (0.011, p-value=0.057) only in week 2 (19 February – 25 February), meaning that in this period vaccine hesitancy grows faster for countries which suspended AstraZeneca vaccine. On the contrary, for the other segments of time it seems that the parallel trend assumption holds, since all other interaction coefficients are not significant.

Table A.1: regression results parallel trend assumption

	VACCINE HESITANCY				
	**WEEK 1 (12-18 Feb.)**	**WEEK 2 (19-25 Feb.)**	**WEEK 3 (26 Feb. - 4 Mar.)**	**WEEK 4 (5 - 11 Mar)**	**WEEK 5 (12-15 Mar.)**
**Trend**	-0.003	0.003	0.003	0.002	-0.019
	(0.004)	(0.005)	(0.004)	(0.006)	(0.013)
**Treated**	-0.069***	-0.090***	0.040*	0.084***	0.119**
	(0.022)	(0.032)	(0.024)	(0.028)	(0.055)
**Treated*Trend**	0.006	0.011*	0.002	-0.008	-0.021
	(0.004)	(0.006)	(0.005)	(0.008)	(0.018)
Observations	10,600	6,523	7,149	4,721	1,884

Robust standard errors in parentheses
*** p<0.01, ** p<0.05, * p<0.1

Notes: Estimation results from equation A1, where the treated (controls) represents individuals living in a country where the AstraZeneca vaccine was (NOT) suspended by 16 March. Control variables are included: gender; education; age; having tested positive to COVID-19; and knowing someone who died because of coronavirus
