# Supplementary material for: Information and vaccine hesitancy: Evidence from the early stage of the vaccine roll-out in 28 European countries
Source: PLoS One. 2022 Sep 21;17(9):e0273555. doi: 10.1371/journal.pone.0273555 (PMC9491558; doi:10.1371/journal.pone.0273555)
Supplement: S2 Appendix — (PDF) [file pone.0273555.s002.pdf]

S2 Appendix – List of suspension dates in the 17 countries

Date	Countries
07/03/21	Austria
11/03/21	Romania, Denmark
12/03/21	Bulgaria
14/03/21	Ireland
15/03/21	France, Germany, Italy, Spain, Luxembourg, Cyprus, Portugal, Slovenia, the Netherlands
16/03/21	Sweden, Latvia

Source: Which countries have stopped using AstraZeneca's COVID vaccine?, March 2021, available here: *https://www.aljazeera.com/news/2021/3/15/which-countries-have-halted-use-of-astrazenecas-covid-vaccine*
