# Supplementary material for: Information and vaccine hesitancy: Evidence from the early stage of the vaccine roll-out in 28 European countries
Source: PLoS One. 2022 Sep 21;17(9):e0273555. doi: 10.1371/journal.pone.0273555 (PMC9491558; doi:10.1371/journal.pone.0273555)
Supplement: S3 Appendix — (PDF) [file pone.0273555.s003.pdf]

S3 Appendix – Percentage of respondents who received at least one dose of a vaccine, by country

	Received at least one dose of the vaccine (%)	Not vaccinated (%)	Not answered or missing (%)
**Austria**	7.72	80.26	12.02
**Belgium**	8.36	79.35	12.29
**Bulgaria**	9.08	73.28	17.64
**Croatia**	6.40	81.87	11.73
**Cyrpus**	4.83	79.48	15.68
**Czechia**	3.98	82.51	13.51
**Denmark**	4.33	83.94	11.73
**Estonia**	9.89	79.34	10.78
**Finland**	5.37	86.48	8.15
**France**	7.15	79.09	13.76
**Germany**	4.47	84.68	10.85
**Greece**	8.18	73.34	18.47
**Hungary**	8.86	77.69	13.45
**Ireland**	7.38	87.98	4.64
**Italy**	7.85	80.52	11.63
**Latvia**	6.25	76.70	17.05
**Lithuania**	7.08	80.95	11.98
**Luxembourg**	4.04	88.51	11.49
**Malta**	18.19	68.80	13.01
**Netherlands**	3.11	83.10	13.79
**Poland**	10.73	68.92	20.35
**Portugal**	5.09	87.61	7.30
**Romania**	20.06	66.63	13.32
**Slovakia**	8.08	77.37	14.56
**Slovenia**	5.84	80.78	13.38
**Spain**	4.45	86.70	8.85

**Sweden**	5.23	84.41	10.36
**UK**	22.09	70.93	6.98
**Whole sample**	**7.87**	**78.80**	**13.33**

Source: Elaboration of our data. Data come from the third wave of the Eurofound "Living, Working and COVID-19"
