# Supplementary material for: Information and vaccine hesitancy: Evidence from the early stage of the vaccine roll-out in 28 European countries
Source: PLoS One. 2022 Sep 21;17(9):e0273555. doi: 10.1371/journal.pone.0273555 (PMC9491558; doi:10.1371/journal.pone.0273555)
Supplement: S4 Appendix — (PDF) [file pone.0273555.s004.pdf]

S4 Appendix. Summary statistics of key variables

VARIABLES	MIN	MAX	MEAN	SD
**Age**	18	100	51.169	14.044
**Female**	0	1	0.630	0.482
**Tertiary**	0	1	0.665	0.472
**Tested positive to COVID-19**	0	1	0.073	0.261
**Death of acquaintance**	0	1	0.096	0.294
**Trust in goverment**	1	10	4.108	2.938
**Trust in EU**	1	10	4.918	2.760
**Trust in healthcare system**	1	10	5.889	2.614
**Trust in pharmaceutical firms**	1	10	4.895	2.541
**Vaccine hesitancy**	0	1	0.229	0.420
**Treated**	0	1	0.591	0.492
**Suspension moment**	0	1	0.128	0.334
**Controversy**	0	1	0.190	0.392
**Sample size**	35390			

Notes: the table shows summary statistics (min, max, mean and standard deviation) of the main dependent and independent variables used in the analysis. In order to better understand how questions were shaped in the Eurofound's LWC survey, the following link could be useful: https://www.eurofound.europa.eu/data/covid-19
