# Supplementary material for: Information and vaccine hesitancy: Evidence from the early stage of the vaccine roll-out in 28 European countries
Source: PLoS One. 2022 Sep 21;17(9):e0273555. doi: 10.1371/journal.pone.0273555 (PMC9491558; doi:10.1371/journal.pone.0273555)
Supplement: S5 Appendix — (PDF) [file pone.0273555.s005.pdf]

S5 Appendix. Mediation analysis

In our analysis we hypothesize that the effect of controversy (and suspension) on vaccine hesitancy may also pass through a mediator, trust. As a result of the controversy (and suspension), trust in institutions (governments and the EU), in the healthcare system and in pharmaceutical firms could have decreased, with a potential knock-out effect on vaccine hesitancy. Therefore, in order to test this reasoning we decided to do a mediation analysis: we will test if the four trust measures can be considered mediators of the controversy (and suspension) effect on vaccine hesitancy. The same control varibales used in the main analysis were used here, with the excelption of factor varibales (age groups). The results of the mediation analysis are reported in the Table A5. The indirect coefficients, which represent the effect of the controversy (and suspension) that passes through trust, are all significant and this means that the four measures of trust are all mediators. We can conclude that, as we mention in the paper, vaccine hesitancy and trust seem to go hand in hand and that a decrease in trust due to the controversy and suspension of the AZ vaccine could have had a negative effect on vaccine hesitancy.

Table A5. Mediation analysis

		**Controversy**	**Suspension**
**Trust in government**	Total effect	0.075***	0.058***
	Direct effect	0.043***	0.029***
	Indirect effect	0.032***	0.029***
**Trust in th EU**	Total effect	0.076***	0.059***
	Direct effect	0.034***	0.021***
	Indirect effect	0.042***	0.038***
**Trust in the healthcare system**	Total effect	0.074***	0.057***
	Direct effect	0.046***	0.037***
	Indirect effect	0.028***	0.020***
**Trust in pharmaceutical firms**	Total effect	0.075***	0.058***
	Direct effect	0.042***	0.031***
	Indirect effect	0.033***	0.027***

Notes: the table reports the results of the mediation analysis done with the SEM command in Stata. The coefficients on the “direct effect” row represent the direct effect of controversy and suspension on vaccine hesitancy. The coefficients on the “indirect effect” row represent instead the effects of controversy and suspension that pass through trust. *** p<0.01, **p<0.05, *p<0.1.
