# Supplementary material for: Information and vaccine hesitancy: Evidence from the early stage of the vaccine roll-out in 28 European countries
Source: PLoS One. 2022 Sep 21;17(9):e0273555. doi: 10.1371/journal.pone.0273555 (PMC9491558; doi:10.1371/journal.pone.0273555)
Supplement: S1 File — (PDF) [file pone.0273555.s006.pdf]

Data Protection Notice on the COVID-19 survey

Updated 12.02.2021

What is Eurofound's e-survey 'Living, working, and Covid-19'?

This online survey aims to capture the most immediate changes in the quality of life and work that occur during the outbreak of the COVID-19 pandemic. A range of questions on topics such as wellbeing, health, the vaccination programmes and sthe quality and use of public services are relevant to people across various age groups and life situations. The survey is designed for a general population of 18 years or older living in the EU Member States, but it is open for people from other countries too.

For further information and updates, please visit Eurofound website: https://www.eurofound.europa.eu/

Who is carrying out the survey?

The survey is being carried out by The European Foundation for the Improvement of Living and Working Conditions (also known as Eurofound)[1].

Eurofound is committed to protecting and respecting your privacy. Personal information collected through this survey will be handled in line with the data protection rules applicable to EU institutions and bodies.[2]

Joint controllership with Facebook

Eurofound allows Facebook to place cookies and tracking pixels on the computer or any other device which you use to complete the survey, regardless of whether you have a Facebook account or not, for the purposes of evaluating the use of the survey and analysing the traffic. Eurofound and Facebook are joint controllers for the processing of personal data resulting from the deployment of this type of technology. The primary controller is:

Facebook Ireland Ltd.
4 Grand Canal Square, Grand Canal Harbour
Dublin 2 Ireland

Facebook Pixel is a part of the Facebook Business Tools. Accordingly, Eurofound and Facebook have entered into an agreement called Facebook Business Tools Terms and Controller Addendum attached to it to determine the respective responsibilities for compliance with the obligations under the applicable data protection law[2] with regard to the joint processing as specified in the applicable product terms of Facebook.

---

[1] *The European Foundation for the Improvement of Living and Working Conditions (Eurofound) is an autonomous body of the European Union, created in 1975 to assist the formulation of future European policy on social and work-related matters.*
[2] *Regulation (EU) 2018/1725 of the European Parliament and of the Council of 23 October 2018 on the protection of natural persons with regard to the processing of personal data by the Union institutions, bodies, offices and agencies and on the free movement of such data, and repealing Regulation (EC) No. 45/2001 and Decision No. 1247/2002/EC, OJ L 295, 21.11.2018, p. 39.*
[2] *Processing of personal data by Facebook is done in compliance with Regulation (EU) 2016/679 of the European Parliament and of the Council of 27 April 2016 on the protection of natural persons with regard to the processing of personal data and*

The Facebook Business Tools Terms and other terms attached to it also sets out the purposes for which the collection and transmission of personal data that constitutes the joint processing takes place. Facebook processes the personal data according to its privacy principles and statements.

Facebook is responsible for enabling your rights under Articles 15-20 of the GDPR with regard to the personal data stored by Facebook after the joint processing. Facebook is also responsible for granting you a right to object to the processing insofar as the joint processing is necessary for the purposes of the legitimate interests pursued by the controller or by a third party, except where such interests are overridden by the interests or fundamental rights and freedoms of the data subject which require protection of personal data, in particular where the data subject is a child[3].

It is also responsible for the security of the Facebook Pixel[4] and for ensuring a notification of a personal data breach to the supervisory authority and for communicating the personal data breach to you[5], insofar as a personal data breaches concerns its obligations under the Controller Addendum.

For more information, mainly on retention periods, amendment of privacy settings on Facebook and rights, please read the Privacy Policy of Facebook.

Please note that in relation to the joint controllership with data deriving from the use of the Facebook Pixel, we only process one type of data: analytics data. Although we do not normally use analytics data to identify you as an individual, you can sometimes be recognised from it, either alone or when combined or linked with other data. In such situations, analytics data can be considered personal data under applicable laws[7] and we will treat as personal data. In addition, analytics data is collected and transmitted to Facebook and its partners (e.g. Instagram).

What personal data will be collected?

You may provide your email in case you consent to be re-contacted for follow-up research. In this case your data will be linked to your email for the purpose of statistical analysis in a pseudonymised form so that any data is not personally identifiable.

The data below will also be collected and processed using cookies, tracking tools and similar technology (including the Facebook Pixel and Google Analytics):

- Http Headers – Anything present in HTTP headers. HTTP Headers are a standard web protocol sent between any browser request and any server on the internet. HTTP Headers include IP addresses, information about the web browser, page location, document, referrer and person using the website;
- Pixel-specific Data – Includes Pixel ID and the Facebook Cookie;
- Button Click Data – Includes any buttons clicked by site visitors, the labels of those buttons and any pages visited as a result of the button clicks;
- Optional Values – Developers and marketers can optionally choose to send additional information about the visit through Custom Data events. Example custom data events are conversion value, page type and more; and

[3] *Article 6(1) (f) of the GDPR.*
[4] *Article 32 of the GDPR.*
[5] *Articles 33 and 34 of the GDPR.*
[7] *Both the EDPR and the GDPR.*

on the free movement of such data, and repealing Directive 95/46/EC (General Data Protection Regulation; hereinafter referred to as 'GDPR').

- Form Field Names – Includes website field names like email, address, quantity, etc., for when you purchase a product or service. We don't capture field values unless you include them as part of Advanced Matching or optional values.

Please note that you may refuse the use of cookies, tracking tool and similar technology. Should you refuse their use, you will not be prevented from participating in the survey.

For more information on how to reject the cookies or delete them on your browser, please read https://www.aboutcookies.org/how-to-control-cookies/ and https://www.aboutcookies.org/how-todelete-cookies/

Who will access your data and how long will it be kept for?

The pseudonymised dataset as well as the data obtained using cookies and similar technology, which will not contain email address or ID numbers, will only be accessed by the research teams within Eurofound. Data collected through the cookies and tracking technology will be transferred to Facebook. No other third parties will have access to your personal data, except if required by law.

If you have agreed to be re-contacted for further research, then your contact details will be kept for up to two years before being securely deleted.

How is your personal data protected?

Several security controls have been put in place to protect your personal data from unauthorised access, use or disclosure. Your data will be stored on servers, located in the European Union hosted by www.soscisurvey.de with access permitted uniquely for authorised research staff.

Your responses will be stored separately from both your email address and the data collected using cookies and similar technology. Your answers will be used solely for research purposes. Your participation, together with your individual responses to the questions, will be kept strictly confidential. The results of the research will be anonymised, used for statistical information only and you will not be identifiable in any published results.

What is the legal basis for processing your personal data?

The entire processing is necessary for the performance of research tasks carried out by Eurofound in the public interest as well as research purposes[6]. Additional processing of your personal data, namely (i) follow-up purposes and (ii) the installation of cookies, pixels and similar technology as well as the processing of personal data resulting therefrom, including transfer of data to Facebook, will be done only if you have given your consent[9]. If you wish to withdraw your consent at any time, please see the 'What are my rights?' section below.

[6] Article 5(1) (a) and 10(2) (j) of the EDPR. [9]
Article 5(1) (d) of the EDPR.

Do we transfer any of your personal data to third countries or international organisations (outside the EU/EEA)?

Our social media, advertising and analytics partners may be located outside the EEA. Data collected through the installation of cookies, tracking and similar technology will only be transferred to a territory outside the EU / EEA area if you consent to the installation of such technology.

Does this processing involve automated decision-making, including profiling?

No decision is taken by Eurofound in the context of this processing activity solely on the basis of an automated processing of your personal data.

What are my rights and how can I exercise them?

Within the limits set by the law, you have the right to access, rectify, erase and/or port your personal data, as well as to restrict or object to the processing of your personal data. Should you wish to exercise any of these rights, please contact

dataprotectionofficer@eurofound.europa.eu

Who can I contact about the survey?

If you have comments or questions, any concerns or a complaint regarding the collection and use of your personal data, please feel free to contact the following:

The European Foundation for the Improvement of Living and Working Conditions (Eurofound)

Wyattville Road, Loughlinstown, Dublin 18, Ireland

Tel: (+353 1) 204 3100; Fax: (+353 1) 282 64 56 / 282 42 09

The Data Controller: The Director of Eurofound, c/o Massimiliano Mascherini

E-mail: esurvey@eurofound.europa.eu

And/or the Data Protection Officer:

E-mail: dataprotectionofficer@eurofound.europa.eu

You may also lodge a complaint before and/or obtain assistance from the following at any time:

European Data Protection Supervisor

Rue Wiertz 60, B-1047 Brussels, Belgium

Tel: (+32) 2 283 1900; Fax: (+32) 2 283 1950

E-mail: edps@edps.europa.eu

Website: http://www.edps.europa.eu/
